# Repetitive transcranial magnetic stimulation over the orbitofrontal cortex for obsessive-compulsive disorder: a double-blind, crossover study

**DOI:** 10.1038/tp.2014.62

**Published:** 2014-09-09

**Authors:** C Nauczyciel, F Le Jeune, F Naudet, S Douabin, A Esquevin, M Vérin, T Dondaine, G Robert, D Drapier, B Millet

**Affiliations:** 1 EA-4712 Behavior and Basal Ganglia Unit, Université de Rennes 1, Rennes, France; 2 Centre Hospitalier Guillaume Régnier, Service Hospitalo-Universitaire de Psychiatrie, Rennes, France; 3 Département de Médecine Nucléaire, Centre Eugène Marquis, Rennes, France; 4 Centre d'Investigation Clinique CIC-P INSERM 0203, Hôpital de Pontchaillou, Centre Hospitalier Universitaire de Rennes et Université de Rennes 1, Rennes, France; 5 Département de Neuroradiologie, Centre Eugène Marquis, Rennes, France

## Abstract

This pilot study was designed to assess the efficacy of low-frequency repetitive transcranial magnetic stimulation (rTMS) over the right orbitofrontal cortex (OFC) by means of a double-cone coil in patients suffering from obsessive-compulsive disorder. We hypothesized that low-frequency stimulation of the OFC would lead to a reduction in clinical symptoms, as measured on the Yale-Brown Obsessive Compulsive Scale (Y-BOCS). A randomized, double-blind, crossover design was implemented with two 1-week treatment periods (active stimulation versus sham stimulation) separated by a 1-month washout period. Concomitantly, a subgroup of patients underwent a positron emission tomography (PET) scan after each stimulation sequence. Statistical analyses compared the Y-BOCS scores at the end of each period. At day 7, we observed a significant decrease from baseline in the Y-BOCS scores, after both active (*P*<0.01) and sham stimulation (*P*=0.02). This decrease tended to be larger after active stimulation than after sham stimulation: −6 (−29, 0) points versus −2 (−20, 4) points (*P*=0.07). Active versus sham PET scan contrasts showed that stimulation was related to a bilateral decrease in the metabolism of the OFC. The OFC should definitely be regarded as a key neuroanatomical target for rTMS, as it is easier to reach than either the striatum or the subthalamic nucleus, structures favored in neurosurgical approaches.

## Introduction

Obsessive-compulsive disorder (OCD) is a frequent and chronic (1-year prevalence: 1.5–2.1%) psychiatric disorder^1^ that is classically treated with selective serotonin reuptake inhibitors (SSRIs) and cognitive behavioral therapy (CBT).^2^ However, this combined treatment is only efficient in 20% patients, with partial remission for 66%. The impairments in social and occupational functioning caused by OCD fully justify the search for new treatments.

For the past 10 years, deep brain stimulation (DBS) has been offered as a treatment for severe and resistant OCD. Nuttin *et al.*^3^ were the first to demonstrate an improvement in OCD brought about by DBS of the ventral portion of the anterior limb of the internal capsule. Other authors have reported similar findings, while the French OCD Stimulation study recorded encouraging results for high-frequency DBS targeting the subthalamic nucleus (STN).^4^ This innovative therapeutic approach has yielded extensive knowledge about the pathophysiology of OCD. For instance, we now know that brain structures such as the anterior cingulate cortex,^5^ orbitofrontal cortex (OFC),^6^ thalamus and striatum,^7^ which are part of the cortico-striato-thalamic loops, are implicated in the dysfunction that leads to OCD. Neuroimaging has also made a valuable contribution to our understanding of OCD pathophysiology: resting-state or active-state studies using functional magnetic resonance imaging or positron emission tomography (PET) have revealed hyperactivation or glucose hypermetabolism of the anterior cingulate cortex, OFC, caudate nucleus and thalamus in OCD patients compared with healthy participants.^8, 9, 10^ The two effective treatments for OCD (CBT and SSRIs) lead to a decrease in the hypermetabolism observed in the prefrontal cortex (PFC), especially the OFC (cortico-striato-thalamic circuit).^11,12^ It turns out that decreased metabolism in this circuit is also a consequence of high-frequency DBS. Nuttin *et al.*^3^ showed, for instance, that DBS of the ventral portion of the interior limb of the internal capsule leads to a decrease in overall brain metabolism and, more specifically, in OFC hypermetabolism. Furthermore, one of the teams in the French OCD Stimulation study^4^ showed that a decrease in scores on the Yale-Brown Obsessive Compulsive Scale (Y-BOCS) was correlated with a decrease in PFC metabolism during STN stimulation.^13^ This result strongly suggests that the efficacy of this technique stems from its capacity to modulate abnormal activity in circuits involving the OFC, anterior cingulate cortex and striatum.^14^ OFC may therefore be a promising treatment target. Considering this set of important results, which strongly suggest that the OFC is a neurofunctional marker of OCD illness, and a potential predictor of response to treatment, we hypothesized that a noninvasive stimulation technique, such as repetitive transcranial magnetic stimulation (rTMS), focused on the OFC might be used as an additional treatment for OCD.

The effect of rTMS on the cortex depends on stimulation frequency. When the motor cortex is the target, frequencies above 1 Hz lead to local excitation, whereas frequencies below 1 Hz trigger inhibition. Stimulation depth seems to depend on the shape of the coil. A figure-of-eight coil only allows for very superficial stimulation (2 cm),^15^ whereas a double-cone coil results in deeper stimulation.^16^

In recent years, the PFC has been the main target in trials of rTMS in OCD, based on the hypothesis of right PFC hypermetabolism in anxiety disorders and depression.^17,18^ In accordance with this hypothesis, several studies have carried out either low-frequency stimulation of the right PFC or high-frequency stimulation of the left PFC, with conflicting findings.^19, 20, 21, 22^

In an open-label study,^23, 24, 25^ Mantovani *et al.*^23^ reported an improvement in OCD after rTMS over the pre-SMA and confirmed these results in a randomized, double-blind study with 18 OCD patients who received 20 low-frequency rTMS sessions over the pre-SMA.^24^ This study was subsequently replicated by Gomes *et al.,*^26^ with 22 patients, with just 10 sessions of low-frequency rTMS over the pre-SMA. These authors observed a long-lasting effect of the treatment over 14 weeks.

Owing to the difficulty of directly stimulating the OFC, which is deeply buried beneath the scalp, this structure has been poorly explored with rTMS. However, in a simple-blind, randomized study targeting the left OFC with low-frequency stimulation from a figure-of-eight coil, Ruffini *et al.*^27^ observed a clinical improvement in the active group compared with the sham stimulation group.

In the present pilot study, we set out to assess the efficacy of low-frequency stimulation of the right OFC in OCD patients using a double-cone coil. We hypothesized that low-frequency stimulation of the OFC would lead to a reduction in clinical symptoms, as measured on the Y-BOCS.^28,29^ We also hypothesized that a double-cone coil would stimulate the OFC more efficiently. As in previous research using high-frequency DBS, we postulated that any improvement would be correlated with a reduction in OFC glucose hypermetabolism.

## Materials and methods

### Design

This study was conducted in the Adult Psychiatry Department of Rennes University Hospital, France. The procedure was based on a randomized, double-blind, crossover design with two 1-week treatment periods (1 week of active stimulation and 1 week of sham stimulation), separated by a 1-month washout period.

Eligible patients were randomly assigned in a 1:1 ratio to one of two groups: one group underwent active stimulation followed by sham stimulation (on–off group) and the other underwent sham stimulation followed by active stimulation (off–on group). After the end of each treatment sequence, the patients underwent a PET scan. The protocol was approved by the ethics committee of Rennes University Hospital, France on 1 June 2010 (no. CPP 10/18-760) and by the French regulatory authority (AFSSAPS; no. 2010-A00294-35). All the patients gave their written informed consent. Because this preliminary study was essentially experimental, the study was not registered on a public trials database.

### Patients

Patients were recruited at the Department of Adult Psychiatry of Rennes University Hospital. The diagnosis of OCD was made using the MINI.^30^ The inclusion criteria for the study were age 18–65 years and a diagnosis of OCD. In addition, all the patients had to have failed to respond to at least two different classes of pharmacological treatment (two SSRIs and clomipramine) used for at least 6 weeks. All patients also had to have failed to respond to CBT. The exclusion criteria were a diagnosis of another psychiatric disorder (except for depressive or anxious disorders), a diagnosis of a significant active medical illness, pregnancy, any history of epilepsy or other neurological illness and any contraindication to TMS or PET. The patients were not allowed to change their medication during the trial.

### Repetitive transcranial magnetic stimulation

rTMS was performed using a Mag2Health × 100 stimulator (Mag2Health, Villennes sur Seines, France). We chose a DB-80 butterfly double-cone coil (Mag2Health) to achieve deeper stimulation of the cortex, including the OFC. For the sham condition, we also used a placebo coil (Medtronic Inc., Minneapolis, MN, USA) that reproduced the sounds, clicks and sensations of actual TMS without the generation of a magnetic field. The patients underwent a visual determination of the motor threshold, defined as the minimum intensity leading to the most prominent abduction of the left abductor pollicus brevis muscle after stimulation of the right motor cortex, holding the coil with the handle pointing backward and laterally at a 45° angle.

The international 10–20 EEG system was used to position the coil over the right OFC, at the right frontopolar 2 (Fp2) electrode site. Ten sessions (two per day over 1 week) were administered using the following parameters: 120% motor threshold, 1 Hz, 1200 pulses per session over the right OFC.

### Randomization and blinding

Randomization was performed without any stratification. Patients were kept blind to the sequence to which they were assigned. Clinical examination was performed by a psychiatrist who was unaware of the patients' stimulation status.

### Outcomes

The primary outcome measure for the study was a change in the Y-BOCS score. Scores on the Clinical Global Impression (CGI)^31^ scale and the Montgomery and Asberg Depression Rating Scale (MADRS)^32^ were used as secondary outcomes. All the assessments were performed before and after each sequence, as well as 1 month after the end of the last session. The side effects of the medical treatment were recorded at each session.

### PET scan

A subgroup of patients underwent two PET scans in Rennes, France (Eugene Marquis Center, Department of Nuclear Medicine) using the same dedicated Discovery ST PET scanner (GEMS, Milwaukee, WI, USA), with an axial field of view of 15.2 cm.

The patients were studied using ^18^FDG PET in a resting state. All the patients were kept on their usual medication. A 144-MBq injection of ^18^FDG was administered intravenously under standardized conditions (in a quiet, dimly-lit room with the patient's eyes and ears open). During the acquisition, the patient's head was immobilized using a head-holder. A cross-laser system was used to achieve stable and reproducible positioning. A three-dimensional emission scan was performed 30 min post injection and after X-ray-based attenuation correction. Following scatter, dead time and random corrections, PET images were reconstructed with three-dimensional-OSEM, yielding 47 contiguous transaxial 3.75-mm thick slices.

At the time of acquisition, the neuroisotopist was blind to the stimulation conditions of each patient. Patients were studied using ^18^FDG PET in a resting state with their eyes open. They underwent two scans: one at the end of the active stimulation sequence, the other at the end of the sham stimulation sequence.

### Statistical analysis

We had not calculated beforehand the number of patients we would need for this pilot study, and we decided to stop inclusions after 3 years. Due to the basic aspect of the issue being addressed, we only performed per-protocol analyses. For all results, data are summarized numerically, with medians (range) for quantitative outcomes (for a small sample size, these parameters provide the least-biased representation of the data) and numbers (percentage) for qualitative outcomes. Wilcoxon's matched-pairs signed-rank test was used to calculate significant differences (two-tailed, *P*<0.05) between the active and sham treatments.

The primary outcome was also analyzed by testing three effects: carryover (physical or psychological effects of the first treatment period still present at the start of the second treatment period), period (difference in stimulation effects between active–sham group and sham–active group) and treatment. In this model, the patient factor corresponded to the random effect (mixed model).

All the statistical analyses were performed with R (R Development Core Team).

### PET scan analysis

The data were analyzed using statistical parametric mapping software (SPM2; Wellcome Department of Cognitive Neurology, London, UK) written in Matlab Version 7 (MathWorks, Sherborn, MA, USA). Statistical parametric maps are spatially extended statistical processes used to characterize specific regional effects in imaging data. They combine the general linear model (to create the statistical map) with the theory of Gaussian fields to make statistical inferences about regional effects.^33^

All patient images were first realigned and spatially normalized to a standard stereotactic space according to the Talairach-Tournoux atlas.^34^ An affine transformation was performed to determine the 12 optimum parameters for registering the brain to the template. The subtle differences between the transformed image and the template were then removed by applying a nonlinear registration method. Finally, the spatially normalized images were smoothed, using an isotropic 12-mm full-width at half-maximum Gaussian Kernel to compensate for interindividual anatomical variability and render the imaging data more normally distributed.

Two contrasts were analyzed:

1. To determine the direct effects of rTMS, we used the ‘population main effect, 2 cond's, 1scan/cond (paired t test)' condition. Clusters of at least 31 contiguous voxels, with a two-tailed *P*-value threshold of 0.005 (corrected for multiple comparisons at cluster level), were considered to be significantly different (as expected and defined by SPM analyses).
2. To validate our *a priori* hypothesis that the benefits of rTMS are related to a decrease in OFC metabolism, we used a general linear ‘multisubject conditions and covariates' model, testing it at each voxel, with the Y-BOCS score as a covariate. This yielded a regression coefficient that was then transformed into *a t*-value. We studied the results for the OFC using the SPM Anatomy toolbox.^35^ We then calculated *t* statistics SPMs, thresholded at *P*=0.005, corrected for multiple comparisons at cluster level, voxel number per cluster *k*>28 (as expected and defined by SPM analyses).

All MNI coordinates were transformed by applying procedures developed by Matthew Brett (http://www.mrc-cbu.cam.ac.uk/Imaging) and reported here, based on the Talairach atlas.

## Results

### Recruitment and baseline data

Supplementary Figure 1 presents the study's flowchart. Between March 2009 and March 2012, a total of 22 patients with moderate-to-severe OCD were recruited. Two patients were included despite an additional diagnosis of psychotic disorder and one despite a diagnosis of anorexia. All three inclusions were deemed to be major deviations from the protocol, and these patients were excluded from all per-protocol analyses. The 19 remaining patients who had begun the trial completed both sequences in a double-blind, crossover and randomized fashion, in accordance with the study protocol. Two patients were lost to follow-up at the final assessment. Medication was held constant throughout the 2 months of the protocol.

At the time of their inclusion in the study, six patients met the criteria for current major depressive disorder. Table 1 provides the baseline data for the 19 patients at inclusion. At the beginning of the study, pharmacological treatment included SSRIs (12/19), antipsychotics (7/19), tricyclic antidepressants (10/19), benzodiazepines (5/19).

### Primary outcome

At day 7, we observed a significant decrease in Y-BOCS scores, compared with baseline, after both active (*P*<0.01) and sham (*P*=0.02) stimulation. This decrease tended to be greater after active stimulation than after sham stimulation: −6 (–29, 0) points versus −2 (−20, 4) points (*P*=0.07).

At day 35, no difference was observed in this decrease from the Y-BOCS baseline between active stimulation and sham stimulation: −1 (−15, 5) points versus 0 (−14, 6) points (*P*=0.94).

We did not detect a significant effect of either carryover or period on these Y-BOCS differences, indicating that the effects of the first treatment period did not persist beyond the washout period.

**Figure 1:**
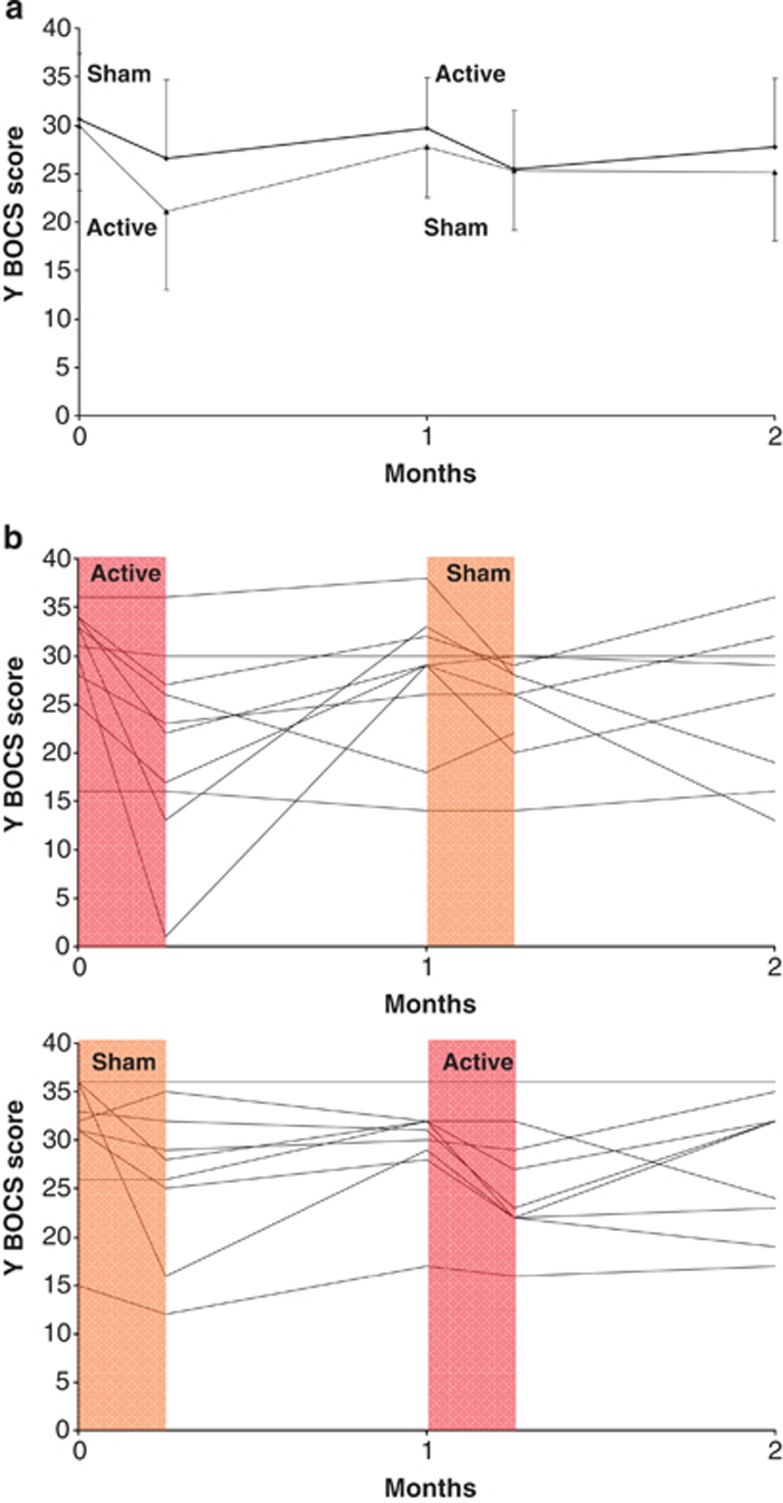
Changes in OCD severity in 19 patients during the crossover study. Data are shown at the time of inclusion in the study (month 0), after the first period of active or sham stimulation (day 7), before (month 1) and after (day 7) the second period of active or sham stimulation and at the end of the study (month 2). (**a**) Shows the mean (s.d.) scores on the Yale-Brown Obsessive Compulsive Scale (Y-BOCS) for the active–sham group (lines) and sham–active group (dashed lines). (**b**) Shows the individual Y-BOCS scores for the active–sham and sham–active groups. The active stimulation period is shown in red and the sham stimulation period in orange. OCD, obsessive-compulsive disorder.

Figure 1 indicates the changes in OCD severity observed in the 19 patients during the crossover study (summary measures and individual data).

### Secondary outcomes

Table 2 sets out the results for all the secondary outcomes. No specific change was observed in MADRS scores.

### PET scan data

**Figure 2:**
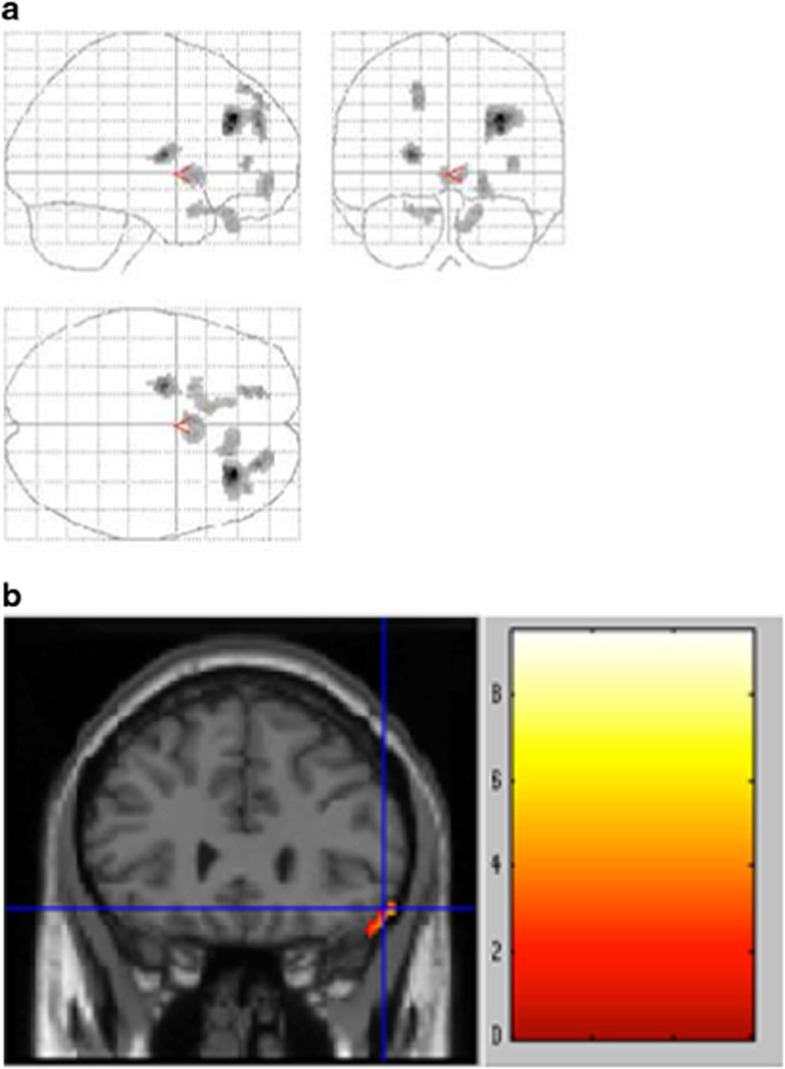
(**a**) Statistical parametric map showing decreased cerebral glucose metabolism in 10 OCD patients treated with rTMS, comparing active stimulation versus sham stimulation conditions. Areas with significant decreases (*P*<0.005, adjusted for multiple comparisons at cluster level) are shown on three telescoped orthogonal views. (**b**) Correlation between metabolic changes induced by rTMS and concomitant clinical improvement (Y-BOCS scores). Whole-brain analysis shows a significant cluster of correlation in the orbitofrontal cortex (Brodmann area 47) with the decrease in the Y-BOCS score.

Ten patients took part in the PET substudy. Areas of significant difference found by comparing the patients in the active and sham conditions are shown in Figure 2a.

In the active stimulation condition, there were a significant decrease in metabolism in the right frontal lobe (superior gyrus (Brodmann area, BA 9), middle gyrus (BA 10), orbital gyrus (BAs 47 and 11)), left anterior cingulate gyrus (BA 25) left frontal lobe (orbital gyrus (BA 11)) and left putamen (Table 3). These results revealed a decrease in the metabolism of the bilateral orbitofrontal lobes, with a more extensive decrease in the right side.

No increase in brain metabolism was found in the active stimulation condition (no cluster significant at *P*<0.005 corrected on cluster level).

When we investigated the neural correlates of the clinical improvement by correlating the decrease in the Y-BOCS score and changes in the PET signal between the two sessions (on versus off, Figure 2b), we found that the decrease in the Y-BOCS score was correlated with a decrease in the metabolic activity of the right OFC (Talairach coordinates +56 +26 −16, BA 47).

### Treatment safety

No serious adverse event was observed during the study. Three patients complained of headache, which resolved without any specific treatment.

## Discussion

### Summary of evidence

Our study revealed a trend toward statistical significance concerning the efficacy of rTMS over the right OFC in OCD at day 7, but no difference was observed a month after the end of the second treatment period. These results are in line with previous findings^27^ showing that low-frequency rTMS over the left OFC produces significant, but time-limited, improvements in OCD patients compared with sham treatment. However, by synchronizing these clinical findings with data yielded by PET scans, we were able to investigate them from a remarkable new perspective. The PET substudy showed that the use of rTMS over the right OFC is associated with a bilateral decrease in the glucose metabolism of that structure. Although different effects of active and sham rTMS on brain metabolism do not strictly exclude the possibility of a placebo effect, it should be noted that the metabolic changes occurred predominantly in front of the coil (BAs 11, 47 and BA 10), and in the right side rather than the left (only BA 11), and a causal relationship therefore seems the more plausible hypothesis. This result suggests that the modulation of the right OFC with rTMS mirrors the metabolic modifications observed with STN DBS in parallel with an improvement in OCD symptomatology (Le Jeune *et al.*^13^). The correlation between the decrease in metabolism and the improvement in OCD could be interpreted as a causal relationship between the treatment, the decrease in OFC metabolism and the therapeutic response. We have previously indicated that the OFC's glucose hyperactivity could represent a therapeutic neurofunctional marker of the disease, with a relationship between a decrease in its activity and the efficacy of high-frequency STN DBS.^13^

Taken together, these results support the hypothesis of a dysfunction of the orbital-subcortical loop in OCD, with two potential basal ganglia targets for brain stimulation techniques (STN or striatum) in resistant and very severe OCD patients, and rTMS over the prefrontal cortex, in particular the OFC, supplementing the classic SSRI and CBT approaches.

Concerning rTMS efficacy, our results underline the theoretical value of stimulating the OFC in OCD, and suggest that a double-cone coil is capable of stimulating this deeply buried structure. Our study is the first to have used such a coil to target the OFC. We can surmise that focusing more accurately on the desired target and using a more appropriate coil would result in a stronger effect. Nevertheless, the positive trend observed in the OCD symptomatology at day 7 was no longer present 1 month later. This time-limited improvement, previously encountered after 10 sessions of 1-Hz rTMS over the OFC^27^ has a very practical implication, for as there is no sustained benefit over time, the usefulness of OFC rTMS in day-to-day clinical practice is therefore limited, given actual stimulation parameters. Nonetheless, rTMS seems a promising tool for exploring the impact of OFC neuromodulation in OCD, as this structure's hyperactivity is strongly implicated in OCD physiopathology. The scientific interest therefore seems greater than the clinical interest, and justifies the use of the per-protocol analysis in the present study, which was designed to determine the biological effect of rTMS in ‘pure' OCD patients.

### Limitations

The present study suffered from low power, which is an endemic problem in the field of neuroscience.^36^ Nevertheless, despite the lack of power in this preliminary study, the trend we observed concerning clinical efficacy was in line with previous research results,^27^ suggesting that OFC rTMS in OCD is a potential treatment that now needs confirmation in an extended study with a larger sample of patients.

Second, there were substantial (and statistically significant) responses in the sham condition group. This may have been, in part, due to the extra attention the OCD patients were given, in line with previous findings (Mansur *et al.*, 2011).^37^

Third, the crossover procedure in this study could be regarded as questionable. Even though our objective was to reproduce the principle of a similar study conducted within the field of OCD,^4^ the duration of the rTMS sequence we administered to OCD patients, as well as the 1-month washout period could be criticized. Moreover, this type of design is prone to unblinding, in that small differences between the sham and active coils may be noticed by the patients, and therefore interfere with their expectations of treatment efficacy, resulting in differential placebo effects across groups. Statistical modeling allowed us to ascertain that there was no significant effect either of carryover or of period on Y-BOCS differences. Nevertheless, these models can suffer from low power, and a closer inspection of Figure 2 suggests that the treatment (be it active or sham) had a greater effect when it was administered in the first period. This argues against the presence of unblinding, as we would have expected a greater improvement for active stimulation after the second session (compared with the first one), when patients had previously experienced sham stimulation and could therefore guess that they were receiving the active treatment. In addition, this aspect of the figure might be in favor of a remanent effect of the last pharmacological change. We acknowledge that the 6-week washout period was quite short, and 12 weeks would have been preferable. Even though the randomized procedure can compensate for this possible confounding factor, it may have resulted in greater variability and thus in a further loss of statistical power.

Moreover, in OCD, a high anxiety state increases obsessive and compulsive symptoms. Treatment onset *per se* may represent a factor for anxiety, particularly in the first period. We did not specifically assess this dimension. However, the randomized order of treatment period lessened the consequences of this lack of data.

### Prospects

On the basis of the parameters we used in our study, our results hold out three main prospects. First of all, this tool could be used in basic research to explore the central role of the OFC in OCD^10,38^ and its functional interconnections with other structures, such as the striatum. These links between neuroanatomical structures could be related to the serotonin dysfunction that is the chief suspect in the pathophysiology of OCD.^39,40^ The specific abnormality of these regions, as well as the ways in which they interact to produce obsessions and compulsions, are as yet unknown. Animal models have recently yielded fresh information about these interconnections, showing that OFC lesions in rats increase compulsive behavior, and that this increase can be prevented by the systemic administration of the SSRI paroxetine. Animal study results also shed new light on the role of a dysfunctional striatal serotonergic system.^41^ In humans, rTMS will allow researchers to explore these issues because it is a noninvasive tool.

A second and equally important point concerns the therapeutic applications arising from our current findings. The parameters for stable improvement now need to be identified, and a neuronavigation system focusing on the OFC, especially the lateral side, might well prove helpful in that respect, as might an increase in the number of stimulation sequences. Furthermore, the use of a more appropriate coil to reach deeper below the scalp would certainly increase the potential efficacy of the technique.

The third issue relates to the generalization of our finding at the individual level, that is, the ability to decipher the prognostic value of this therapeutic approach for a given patient. In this regard, it would be well worth conducting a diagnostic accuracy study of OFC hyperactivity.

To confirm these promising findings and to address the above mentioned limitations, we are planning to conduct a randomised, single-blind, controlled trial of active OFC rTMS versus sham OFC rTMS with a blinded outcome assessment. Based on this study, we predict an effect size of nearly 0.5. The statistical analysis performed on the main endpoint will be conducted using a two-sided test with a Type I error of 5%. Based on these hypotheses, the sample size, computed to guarantee a power of 80%, will have to be 60 patients per group (that is, a total of 120 patients).

## Conclusion

The results of this preliminary study suggest that the OFC is a possible neuroanatomical target for OCD treatment, especially rTMS. Over the past 20 years, brain stimulation techniques have shown themselves to be genuine alternatives to classic treatments such as CBT and SSRIs. If confirmed, they may prove at least as effective as SSRIs, and reflect a rational approach to the disease based on the hypothesis of a dysfunctional PFC-basal ganglia circuit. Moreover, the results of this study allow us to regard the OFC as an additional neuroanatomical target, and one that is easier to reach than the striatum or the STN favored in neurosurgical approaches. Larger trials using rTMS over the OFC should be carried out to confirm our results and to confirm rTMS as an effective and complementary treatment for OCD.

## Figures and Tables

**Table 1:** Baseline data for the 19 patients at inclusion

	*All patients*	*On*–*off group*	*Off*–*on group*
Age	39 (24, 56)	40 (24, 56)	39 (26, 56)
Sex (women)	15 (79%)	8 (80%)	7 (78%)
Age at onset	18 (7, 48)	18 (14, 32)	15 (7, 48)
			
*Y-BOCS*	32 (15, 36)	32 (16, 36)	32 (15, 36)
Y-BOCS obsession subscale	16 (8, 18)	16 (8, 18)	16 (11, 18)
Y-BOCS compulsion subscale	16 (0, 20)	17 (0, 20)	16 (1, 19)
			
CGI	6 (5, 7) (NA=6)	7 (5, 7) (NA=4)	6 (5, 7) (NA=2)
MADRS	12 (3, 35) (NA=3)	22 (3, 35) (NA=1)	10 (6, 16) (NA=2)
			
*Current medication*	(NA=2)	(NA=2)	
SRI	12 (70%)	5 (63%)	7 (78%)
SNRI	1 (6%)	1 (13%)	0 (0%)
Tricyclic antidepressant	10 (60%)	6 (75%)	4 (44%)
Antipsychotics	7 (41%)	1 (13%)	6 (67%)
Mood stabilizer	2 (11%)	1 (13%)	1 (11%)
Antihistamine	1 (6%)	0 (0%)	1 (11%)
Benzodiazepine	5 (26%)	3 (38%)	2 (22%)

Abbreviations:

CGI, Clinical Global Impression Scale; MADRS, Montgomery and Asberg Depression Rating Scale; NA, number of missing data; SNRI, serotonin and norepinephrine reuptake inhibitors; SRI, serotonin reuptake inhibitors; Y-BOCS, Yale-Brown Obsessive Compulsive Scale.

For all results, data are summarized numerically, with medians (range) for quantitative outcomes and numbers (percentage) for qualitative outcomes.

**Table 2:** Changes in OCD severity, clinical impressions and depression

	*Active period*	*Sham period*	P*-value*
*Change after treatment (day 7)*			
** ***Y-BOCS*	−6 (−29, 0)	−2 (−20, 4)	0.07
** **Y-BOCS obsession subscale	−2 (0, 16)	−1 (−2, 10)	0.08
** **Y-BOCS compulsion subscale	−3 (0, 13)	−1 (−3, 10)	0.22
CGI	3 (1, 4) (NA=3)	3 (1, 5) (NA=1)	0.35
MADRS (change)	−2 (−28, 2) (NA=2)	0 (−19, 7) (NA=5)	0.64
			
*Change at follow-up (day 35: 1 month after treatment)*			
** ***Y-BOCS*	−1 (−15, 5)	0 (−14, 6) (NA=2)	0.94
** **Y-BOCS obsession subscale	0 (−2, 7)	0 (−5, 8) (NA=2)	0.75
** **Y-BOCS compulsion subscale	0 (−3, 8)	0 (−1, 6) (NA=2)	0.59
CGI	3 (1, 4) (NA=1)	3 (1, 5) (NA=5)	0.74
MADRS	0 (−21, 10) (NA=3)	−1 (−6, 13) (NA=6)	0.69

Abbreviations:

CGI, Clinical Global Impression Scale; MADRS, Montgomery and Asberg Depression Rating Scale; NA, number of missing data; OCD, obsessive-compulsive disorder; Y-BOCS, Yale-Brown Obsessive Compulsive Scale.

For all results, data are summarized numerically, with medians (range) for quantitative outcomes and numbers (percentage) for qualitative outcomes.

**Table 3:** Regions with decreased glucose metabolism after active rTMS stimulation in 10 OCD patients (*P*<0.005, multiple comparison corrected on cluster level, voxel number per cluster *k*>31)

*Region*	*Talairach coordinates*			Z*-value*	*Voxel number*
	*X*	*Y*	*Z*		
Right frontal lobe, middle gyrus, BA 9	30	34	32	4.50	424
Right frontal lobe, middle gyrus, BA 10	24	52	20	4.24	303
Left putamen	−22	−6	10	4.05	110
Right frontal lobe, middle gyrus, BA 10	20	52	−10	3.39	86
Left cingulate gyrus, BA 25	−2	10	−2	3.38	105
Right orbital gyrus, BA 47	18	32	−24	3.30	94
Right orbital gyrus, BA 11	12	32	−30	3.14	94
Left orbital (rectal) gyrus, BA 11	−10	22	−22	3.22	98

Abbreviations: BA, Brodmann area; OCD, obsessive-compulsive disorder; rTMS, repetitive transcranial magnetic stimulation. The results are classified according to *z*-score values.

## Notes

This study was supported by a grant from the Fondation Pierre Deniker. The funders had no role in study design, data collection and analysis, decision to publish, or preparation of the manuscript. All authors declare that (1) BM has relationships (consultancy and travel/accommodation expenses covered/reimbursed) with Janssen, BMS, Otsuka, Lundbeck, Lilly, Servier, Astra Zeneca, Medtronic and Syneïka, and has received grants for research from Medtronic, Lilly and Astra Zeneca within the previous 3 years; FN has relationships (board membership or travel/accommodation expenses covered/reimbursed) with Servier, BMS, Lundbeck and Janssen, which might have an interest in the work submitted, within the previous 3 years; (2) CN's spouse is the director of Sineika; BM's spouse is an employee of Janssen; and (4) none of the authors has any nonfinancial interests that may be relevant to the submitted work.
