# Supplementary figures and images for: Repetitive transcranial magnetic stimulation over the orbitofrontal cortex for obsessive-compulsive disorder: a double-blind, crossover study
Source: Transl Psychiatry. 2014 Sep 9;4(9):e436–. doi: 10.1038/tp.2014.62 (PMC4203001; doi:10.1038/tp.2014.62)

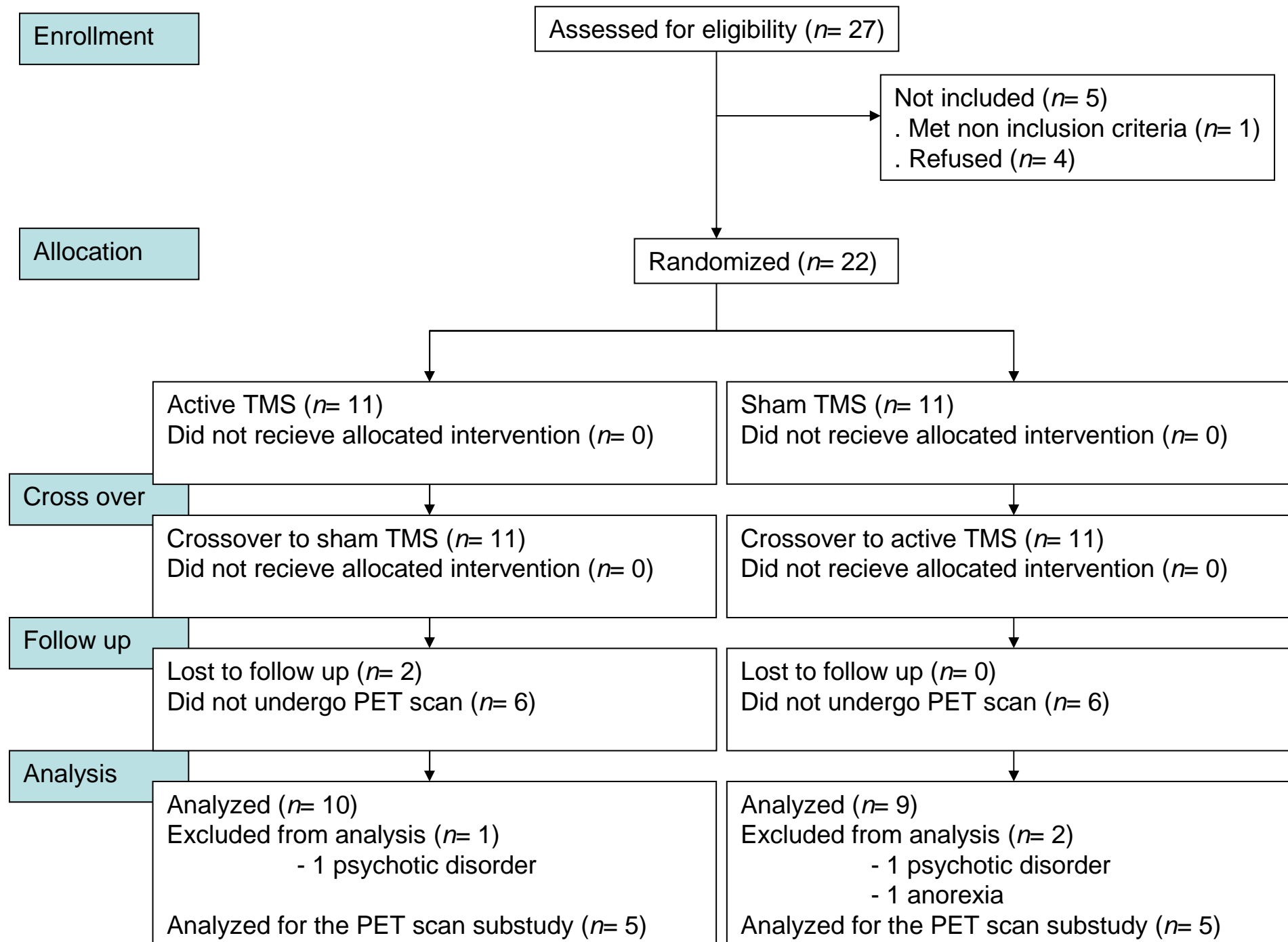

Supplement: Supplementary Figure 1 [file tp201462x1.pdf]
