# Supplementary material for: Repetitive transcranial magnetic stimulation over the orbitofrontal cortex for obsessive-compulsive disorder: a double-blind, crossover study
Source: Transl Psychiatry. 2014 Sep 9;4(9):e436–. doi: 10.1038/tp.2014.62 (PMC4203001; doi:10.1038/tp.2014.62)
Supplement: Supplementary Figure Legend [file tp201462x2.doc]

e-Figure 1: CONSORT 2010 flowchart outlining the design and conduct of the clinical study.
